# The Life of Man

**Published:** 1883-09

**Authors:** 


### THE LIFE OF MAN.

Man, born of woman is of a few days and no teeth. And indeed it would be money in his pocket sometimes if he had less of either. As for his days, he wasteth one third of them; and as for his teeth, he has convulsions when he cuts them, and as the last one comes through, lo, the dentist is twisting the first one out, and the last end of that man's jaw is worse than the first, being full of porcelain and a roof-plate built to hold blackberry seeds.—*Burlington Hawkeye.*

